# Daphnetin alleviates inflammation and promotes autophagy via the AMPK/mTOR pathway in gouty arthritis

**DOI:** 10.1002/ccs3.70011

**Published:** 2025-04-28

**Authors:** Zhiyong Liu, Aichun Chu, Zhiqian Bai, Chao Yang

**Affiliations:** ^1^ Department of Rheumatology and Immunology Renmin Hospital Wuhan University Wuhan Hubei China; ^2^ Department of Orthopedics Maternal and Child Health Hospital of Hubei Province Wuhan China

**Keywords:** autophagy, daphnetin, gouty arthritis, monosodium urate crystal

## Abstract

Gouty arthritis (GA) is an inflammatory disease resulting from monosodium urate (MSU) crystal deposition in joints and surrounding tissues. Daphnetin (DAP) is a coumarin derivative with potent anti‐inflammatory activity. Nonetheless, whether DAP can protect against MSU‐induced acute GA remains unclarified. In this study, C57BL/6 mice were injected intra‐articularly with MSU crystal suspension to induce acute GA. THP‐1 cells were stimulated with MSU to mimic the microenvironment of GA in vitro. Hematoxylin–eosin staining was conducted to observe the pathological changes in mouse synovial tissues. ELISA and RT‐qPCR were employed for inflammatory cytokine level determination. Immunofluorescence staining was performed to estimate LC3 expression in THP‐1 cells. Western blotting was used for protein expression analysis. The results showed that DAP pretreatment mitigated MSU‐elicited ankle joint swelling and synovial damage in mice. Moreover, DAP hindered proinflammatory factor expression and promoted autophagy in MSU‐stimulated GA mice and THP‐1 cells. Mechanistically, DAP induced AMPK activation and mTOR inactivation. Blocking AMPK signaling counteracted DAP‐mediated effects on inflammation and autophagy in MSU‐stimulated THP‐1 cells. In conclusion, DAP prevents MSU‐elicited GA by alleviating inflammation and enhancing autophagy via AMPK/mTOR signaling transduction.

## Graphical abstract

Daphnetin, a coumarin derivative, regulates the AMPK/mTOR signaling pathway to inhibit inflammation and promote autophagy in monosodium urate‐induced mice and THP‐1 cells, thereby suppressing MSU‐induced gouty arthritis.

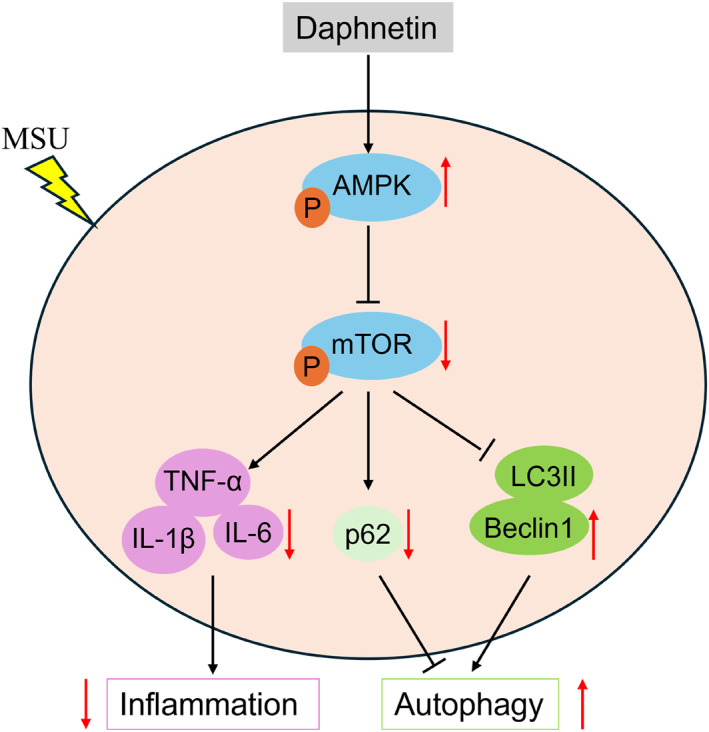

## 1 INTRODUCTION

Gouty arthritis (GA) is an inflammatory disease resulting from monosodium urate (MSU) crystal deposition in joints and surrounding tissues.^1^ The prevalence of GA has been increasing in recent years due to the aging population and lifestyle changes.^2^ The main clinical manifestations include joint pain, redness, swelling, and heat. In severe cases, it can lead to limited movement and joint deformity, seriously impairing the quality of life of affected patients.^3^ At present, GA patients are often prescribed colchicine, nonsteroidal anti‐inflammatory drugs (NSAIDs), or glucocorticoids for pain relief.^4^ Nonetheless, long‐term administration of these drugs can cause side effects, such as renal toxicity, hepatotoxicity, and allergic reactions, which limit their application.^5^, ^6^ Therefore, it is imperative to identify new effective therapeutic agents for the disease.

Autophagy is a cellular self‐degradation process pivotal for the maintenance of intracellular homeostasis.^7^ Evidence suggests that autophagy inhibits inflammasome activation, subsequently reducing inflammatory cytokine production and promoting damaged tissue repair.^8^ Dysfunctional autophagy is closely related to the pathogenesis and development of various diseases.^9^ Importantly, studies have shown that autophagy also acts as a critical player in GA. For instance, Yuan et al. demonstrated that PPP21 ameliorates MSU‐evoked acute GA by inducing autophagy to suppress NLRP3 inflammasome activation.^10^ Liu et al. proposed that promoting autophagy can mitigate GA by hindering PI3K/Akt/mTOR signaling transduction.^11^ Many signaling pathways are involved in the regulation of autophagy, including the AMP‐activated protein kinase/mechanistic target of rapamycin (AMPK/mTOR) pathway. In response to nutrient deprivation, autophagy is initiated by AMPK activation and mTOR inactivation.^12^ A previous study indicated that Zisheng Shenqi Decoction could ameliorate MSU‐triggered GA by regulating the AMPK/mTOR signaling pathway to promote autophagy.^13^

Daphnetin (7,8‐dihydroxy‐coumarin, DAP) is a coumarin derivative found in the *Daphne* genus. Studies have revealed that DAP is a bioactive substance with copious activities, including anti‐inflammatory, antioxidant, analgesic, and neuroprotective activities.^14^, ^15^ Moreover, previous evidence has suggested the beneficial effects of DAP in inflammatory diseases. For instance, DAP reduces the production of proinflammatory factors and alleviates oxidative stress in ulcerative colitis.^16^ Fan et al. demonstrated the protective effects of DAP against PM2.5‐elicited airway inflammation in a mouse model.^17^ Importantly, previous reports have indicated that DAP has significant therapeutic effects on collagen‐triggered arthritis.^18^, ^19^ DAP also exhibits antiarthritic and chondroprotective effects in the model of osteoarthritis.^20^ Nevertheless, its specific functions in MSU‐induced GA remain unclarified.

Herein, we intend to figure out the functions of DAP in GA using a mouse model and an in vitro cell model. We hypothesized that DAP might exert an antiarthritic effect in GA by regulating inflammation and autophagy via AMPK/mTOR signaling.

## 2 MATERIALS AND METHODS

### 2.1 Preparation of DAP and MSU suspension

DAP (99.86% purity, MedChemExpress, Shanghai, China) was dissolved in 1% DMSO and diluted with phosphate‐buffered saline (PBS).

### 2.2 Animals

Male C57BL/6 mice (7–8 weeks, from Cavens, Changzhou, China) were housed in a specific pathogen‐free environment under 12‐h light/dark cycles with controlled temperature (23 ± 2°C) and humidity (55%–60%) and had free access to food and water. They were allowed to acclimate for a week before experiments. All animal studies were conducted as per the Guide for the Care and Use of Laboratory Animals, with approval from the ethics committee of Wuhan Myhalic Biotechnology Co., Ltd (approval number: HLK‐202305397; approval date: May 26, 2024).

### 2.3 Animal grouping and treatment

Twenty‐four mice were randomly assigned to 4 groups (*n* = 6/group): (1) control group, (2) MSU (model) group, (3) MSU + 10 mg/kg DAP (DAP‐10) group, and (4) MSU + 20 mg/kg DAP (DAP‐20) group. Mice in the DAP‐treated groups were administered 10 or 20 mg/kg DAP for 7 consecutive days via oral gavage. The doses of DAP were determined based on previous reports.^21^, ^22^ One hour after DAP administration on the seventh day, all mice, except those in the control group, were injected intra‐articularly with MSU crystal suspension (0.5 mg in 25 μL PBS) into the left ankle joint to induce acute GA.^23^ The control mice received 25 μL PBS alone. To evaluate ankle joint swelling, the ankle circumference was measured using an electronic caliper at different time points after MSU injection (4, 8, 12, and 24 h). The increase in circumference between adjacent time points was evaluated.

### 2.4 Sample collection

Twenty‐four hours after MSU injection, all mice were euthanized with an overdose of pentobarbital sodium (100 mg/kg). Blood samples were collected and centrifuged to obtain the serum. Ankle joint tissues were harvested for histological analysis, ELISA, and western blotting.

### 2.5 H&E staining

Fresh ankle joint tissues were fixed with 4% paraformaldehyde, decalcified with 10% EDTA, embedded in paraffin, and cut into sections (4 μm thick). After deparaffinization and rehydration, the sections were stained with hematoxylin and eosin (Solarbio, Beijing, China) as per the manufacturer's protocols. The staining results were observed under a light microscope (Leica Microsystems, Shanghai, China).

### 2.6 Cell culture and treatment

THP‐1 cells (WheLab, Shanghai, China) were cultured in RPMI‐1640 medium (Gibco, Grand Island, NY) containing 10% fetal bovine serum (Gibco) and 0.05 mM β‐mercaptoethanol (Gibco) in a humidified incubator (5% CO_2_, 37°C). THP‐1 cells were treated with 50 nM PMA for 24 h to induce differentiation into macrophages.

To estimate the role of DAP in MSU‐induced GA in vitro, THP‐1 macrophages were pretreated with 500 ng/mL lipopolysaccharide (LPS) for 4 h, then treated with various concentrations of DAP (10, 20, and 40 μM) for 1 h, and stimulated with 200 μg/mL MSU for 5 h.^2^

### 2.7 CCK‐8 assay

For cell viability assessment, THP‐1 cells were inoculated into 96‐well plates (5 × 10^3^/well). After the indicated treatments, cells in each well were incubated with 10 μL cell counting kit‐8 (CCK‐8) reagent (Beyotime, Shanghai, China) for an additional 1 h. Detection of absorbance at 450 nm was performed using a microplate reader (Thermo Scientific, Waltham, MA).

### 2.8 Evaluation of LDH release

Lactate dehydrogenase (LDH) release in the supernatant of the cell culture medium was evaluated using an LDH Assay Kit (Beyotime). Briefly, 120 μL of the supernatant was added to a 96‐well plate. Then, 60 μL LDH solution was added to each well and incubated for 30 min without light. The absorbance was assessed at 490 nm using a microplate reader (Thermo Scientific).

### 2.9 Western blotting

THP‐1 cells or mouse synovial tissues were primed using RIPA lysis buffer (Beyotime) to extract proteins. A BCA Assay Kit (Beyotime) was employed for protein concentration evaluation. Afterward, protein samples (20 μg) were separated by 10% or 12% SDS‐PAGE and blotted onto PVDF membranes (Beyotime). After treatment with QuickBlock™ Western Blocking Buffer (Beyotime) for 10 min, the membranes were incubated overnight at 4°C with the indicated primary antibodies (Table 1) and rinsed with PBS three times before incubation with the secondary antibody (1:2000, ab288151, Abcam) for 1 h. Lastly, the blots were visualized using BeyoECL Plus (Beyotime) and analyzed using the ImageJ software.

**TABLE 1:** Primary antibodies used in western blotting.

Target	Cat. no.^a^	Dilution
LC3	ab192890	1:2000
Beclin1	ab207612	1:2000
p62	ab109012	1:10,000
AMPK	ab207442	1:1000
mTOR	ab134903	1:10,000
p‐AMPK	ab133448	1:1000
p‐mTOR	ab109268	1:1000
GAPDH	ab9485	1:2500

^a^ All from Abcam, Shanghai, China.

### 2.10 RT‐qPCR

Total RNA isolation from THP‐1 cells was achieved using TRIzol reagent (Beyotime). The iScript cDNA Synthesis Kit (Bio‐Rad, Hercules, CA) was employed for RNA reverse transcription into cDNA. RT‐qPCR was conducted using SYBR Green Master Mix (Yeason, Shanghai, China) on a CFX96 Touch Real‐Time PCR Detection System (Bio‐Rad). With GAPDH as normalization, the relative cytokine mRNA level was computed using the 2^−ΔΔCt^ method. The primer sequences are listed in Table 2.

**TABLE 2:** Primer sequences used in RT‐qPCR.

Gene	Forward (5′‐3′)	Reverse (5′‐3′)
IL‐6	TACCACTTCACAAGTCGGA	AATTGCCATTGCACAACTC
IL‐1β	GCCTCAAAGGAAAGAATCTATACC	CTTGGGATCCACACTCTCC
TNF‐α	TTCTCATTCCTGCTTGTGG	TTGGGAACTTCTCATCCCT
GAPDH	ACTCTTCCACCTTCGATGC	CCGTATTCATTGTCATACCAGG

### 2.11 ELISA

The concentrations of proinflammatory cytokines in the serum samples, mouse synovial tissues, or THP‐1 cells were determined using commercial ELISA kits (CUSABIO, Wuhan, China) as per the manufacturer's protocols.

### 2.12 IF staining

THP‐1 cells were fixed with 100% ice‐cold methyl alcohol for 5 min, permeabilized with 0.1% Triton X‐100 for 10 min, and blocked with 10% normal goat serum for 1 h. Afterward, the cells were incubated with an anti‐LC3 primary antibody (1 μg/mL, ab192890, Abcam) overnight at 4°C and then further incubated with the secondary antibody (2 μg/mL, ab150077, Abcam) for 1 h away from light. DAPI (Solarbio) was employed to label the nucleus. Images were captured using a fluorescence microscope (Leica Microsystem).

### 2.13 Molecular docking

The molecular docking method was used to evaluate the interaction between DAP and AMPK. The 3D crystal structure of AMPK (PDB ID: 5UFU) was obtained from the Protein Data Bank (https://www.rcsb.org). The 2D SDF format of DAP was downloaded from PubChem. For docking analysis, the protein and molecule files were converted to PDBQT format, with all water molecules removed and polar hydrogen atoms added. Molecular docking calculations were performed using AutoDock Vina 1.2.2 (https://vina.scripps.edu/).

### 2.14 Statistical analysis

Data are presented as the mean ± standard deviation. Differences among groups were evaluated by one‐way ANOVA using the GraphPad Prism 8.0.2 software (GraphPad, San Diego, CA). Tukey's post hoc test was used for all analyses. *p* < 0.05 defined statistical significance.

## 3 RESULTS

### 3.1 DAP attenuates MSU‐induced articular swelling in GA mice

**FIGURE 1:**
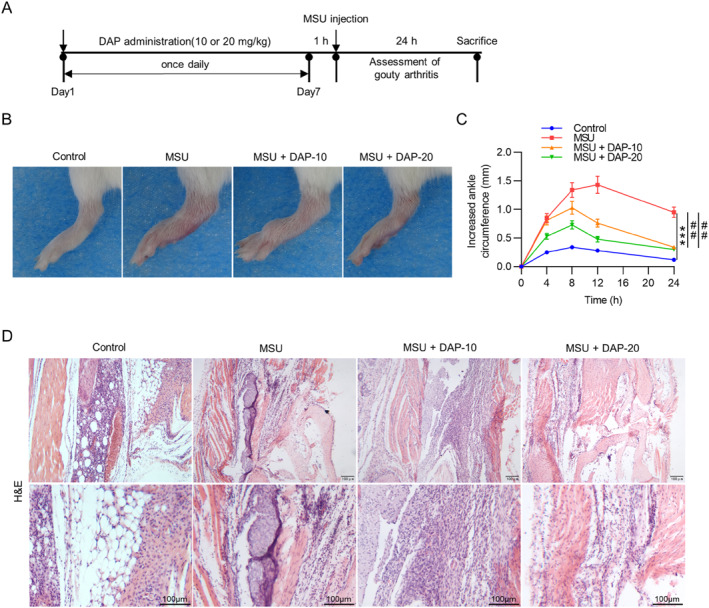
Daphnetin attenuates MSU‐induced articular swelling in gouty arthritis mice. (A) The scheme for the experimental model. (B) Representative images of ankles 24 h after MSU injection. (C) Measurement of increased ankle circumference at different time points after MSU injection. (D) Representative images of H&E staining showing histopathological changes in the synovial tissues of the ankle joint. *n* = 6/group. MSU, monosodium urate. ***p < 0.001 versus control; ##*p* < 0.01 versus MSU.

To determine the effect of DAP on GA, mice were administered DAP (10 or 20 mg/kg) followed by injection of MSU into the ankle joint to induce acute GA (Figure 1A). As shown in Figures 1B–C, compared with the control group, the MSU‐induced model group exhibited significant ankle swelling, which peaked 12 h after MSU crystal injection, indicating the successful establishment of the GA mouse model. In contrast, DAP pretreatment markedly prevented MSU‐induced ankle swelling in mice (Figures 1B–C). Moreover, hematoxylin–eosin (H&E) staining was conducted to estimate the histopathological changes in synovial tissues. Notably, there was severe synovial edema and inflammatory cell infiltration in the MSU group compared to the control group. Nonetheless, these pathological changes caused by MSU were prominently alleviated in DAP‐treated groups (Figure 1D), suggesting the protective effect of DAP against MSU‐triggered GA in mice.

### 3.2 DAP alleviates inflammatory response in MSU‐induced mice

**FIGURE 2:**
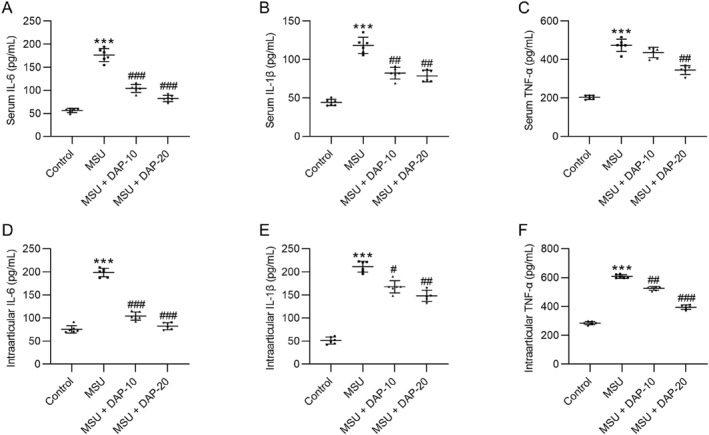
Daphnetin alleviates the inflammatory response in MSU‐induced mice. (A–C) ELISA for determining serum levels of IL‐6, IL‐1β, and TNF‐α in each group. (D–F) ELISA for evaluating the concentrations of IL‐6, IL‐1β, and TNF‐α in the synovial tissues of the ankle joint. *n* = 6/group. MSU, monosodium urate. ***p < 0.001 versus control; #*p* < 0.05, ##*p* < 0.01, ###*p* < 0.001 versus MSU.

Subsequently, we estimated whether DAP impacted MSU‐induced inflammation in GA by measuring serum and intracellular levels of proinflammatory cytokines in each group. As expected, the serum levels of IL‐6, IL‐1β, and TNF‐α were remarkably elevated after MSU injection (Figures 2A–C), confirming that MSU contributed to the inflammatory response in GA. Notably, DAP dose‐dependently reduced the concentrations of these proinflammatory factors in the serum samples of MSU‐induced mice (Figures 2A–C). Similar changes in the levels of these proinflammatory factors were observed in the synovial tissues of each group (Figures 2D–F). Taken together, DAP exerts an anti‐inflammatory effect on MSU‐induced GA.

### 3.3 DAP activates autophagy in MSU‐induced mice

**FIGURE 3:**
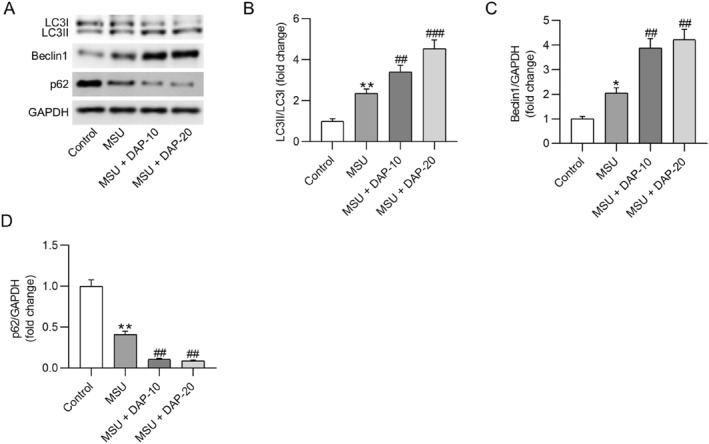
Daphnetin activates autophagy in MSU‐induced mice. (A) Western blotting for estimating autophagy‐related protein levels in mouse synovial tissues. (B–D) Quantitative results of western blotting. *n* = 6/group. MSU, monosodium urate. *p < 0.05, **p < 0.01 versus control; ##*p* < 0.01, ###*p* < 0.001 versus MSU.

Autophagy has been indicated to affect the inflammatory response in GA. We also examined DAP's effect on autophagy in GA. Western blotting showed that MSU activated autophagy in synovial tissue, as evidenced by an increased LC3II/LC3I ratio and Beclin1 expression and a decreased p62 expression (Figures 3A–D). DAP markedly enhanced the LC3II/LC3I ratio and Beclin1 expression and reduced p62 expression in the synovial tissue of GA mice (Figures 3A–D). These data indicated that DAP could enhance autophagy in the MSU‐induced mouse model.

### 3.4 DAP reduces proinflammatory factor levels in MSU‐stimulated THP‐1 cells

**FIGURE 4:**
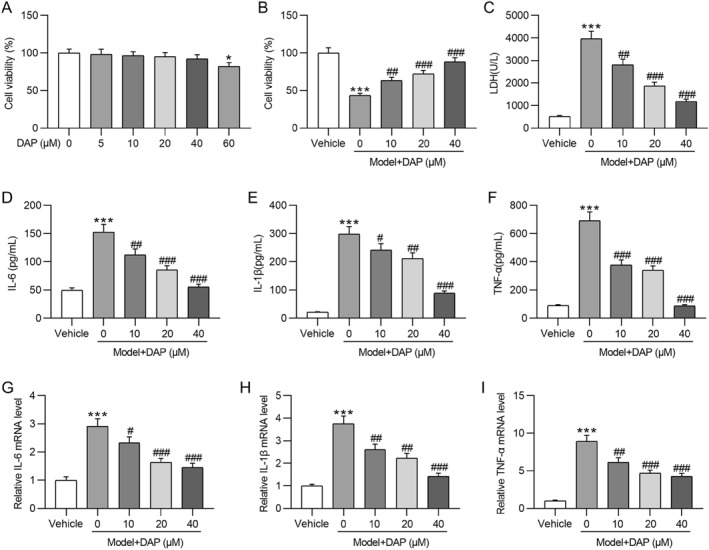
Daphnetin reduces proinflammatory factor levels in MSU‐stimulated THP‐1 cells. (A) CCK‐8 assay for evaluating THP‐1 cell viability under treatment with various concentrations of DAP for 24 h. (B) CCK‐8 assay for assessing THP‐1 cell viability under treatment with vehicle, MSU, or MSU plus DAP (10, 20, or 40 μM). (C) Evaluation of lactate dehydrogenase release in the culture medium of each group. (D–F) ELISA for determining proinflammatory cytokine concentrations in indicated THP‐1 cells. (G–I) RT‐qPCR analysis of cytokine mRNA levels in THP‐1 cells of each group. DAP, daphnetin; MSU, monosodium urate. *p < 0.05 versus DAP (0 μM); ***p < 0.001 versus vehicle; #*p* < 0.05, ##*p* < 0.01, ###*p* < 0.001 versus model + DAP (0 μM).

Furthermore, in vitro experiments were carried out to verify the DAP effect on MSU‐triggered inflammation. We first assessed the cytotoxicity of DAP to THP‐1 cells under normal conditions. As depicted by the CCK‐8 assay, 60 μM DAP remarkably impaired THP‐1 cell viability, whereas DAP at concentrations of 5–40 μM showed no significant cytotoxic effects (Figure 4A). Thus, DAP concentrations of 10, 20, and 40 μM were selected for subsequent experiments. Figure 4B revealed that MSU stimulation markedly reduced THP‐1 cell viability, whereas DAP preconditioning dose‐dependently abated this effect caused by MSU. Likewise, MSU‐triggered LDH release was prominently counteracted by DAP treatment (Figure 4C), suggesting the cytoprotective effect of DAP. Consistent with animal experiments, the results demonstrated that MSU elicited the secretion of proinflammatory cytokines (IL‐6, IL‐1β, TNF‐α) in THP‐1 cells (Figures 4D–F). In contrast, DAP dose‐dependently reduced the concentrations of these cytokines in MSU‐stimulated THP‐1 cells (Figures 4D–F). Similarly, DAP preconditioning impeded the MSU‐triggered increase in cytokine mRNA levels in THP‐1 cells, as demonstrated by RT‐qPCR (Figures 4G–I), confirming the anti‐inflammatory activity of DAP.

### 3.5 DAP enhances autophagy in MSU‐treated THP‐1 cells

**FIGURE 5:**
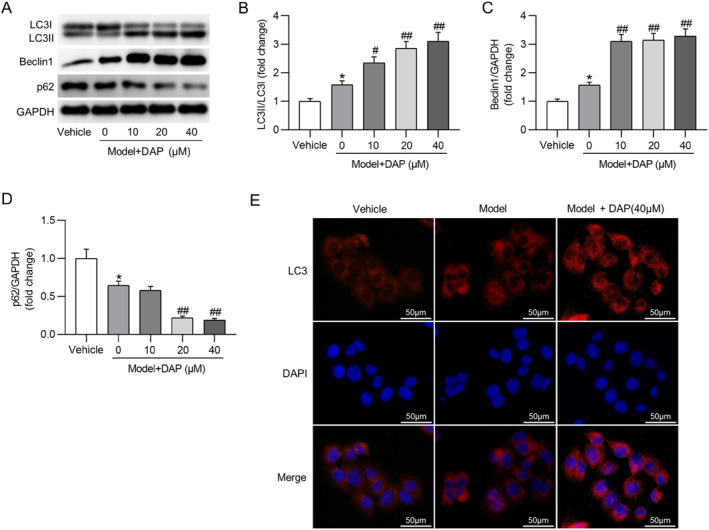
DAP enhances autophagy in monosodium urate‐treated THP‐1 cells. (A) Western blotting showing protein levels of autophagy‐associated markers in indicated THP‐1 cells. (B–D) Quantification of relative protein levels. (E) Representative images of IF staining for detecting LC3 expression in THP‐1 cells. DAP, daphnetin. *p < 0.05 versus vehicle; #*p* < 0.05, ##*p* < 0.01 versus model + DAP (0 μM).

As shown in Figures 5A–C, DAP markedly elevated the LC3II/LC3I ratio and promoted Beclin1 expression in THP‐1 cells under MSU stimulation. At the same time, compared to that in the model group, the p62 expression in DAP‐treated groups was prominently decreased (Figures 5A,D). Immunofluorescence (IF) staining also displayed that DAP pretreatment facilitated LC3 expression in THP‐1 cells under MSU stimulation (Figure 5E). The above data revealed that DAP enhanced autophagy in MSU‐stimulated THP‐1 macrophages.

### 3.6 DAP regulates AMPK/mTOR signaling transduction

**FIGURE 6:**
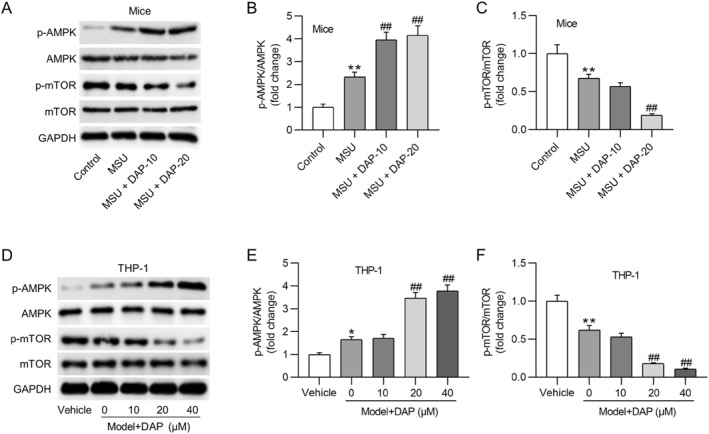
DAP regulates AMPK/mTOR signaling transduction. (A–C) Western blotting showing (p)‐AMPK and (p)‐mTOR protein expression in the mouse synovial tissues of each group. (D–F) Western blotting depicting (p)‐AMPK and (p)‐mTOR protein expression in THP‐1 cells. DAP, daphnetin. *p < 0.05, **p < 0.01 versus vehicle; ##*p* < 0.01 versus model + DAP (0 μM).

To explore the potential mechanism by which DAP protected against MSU‐evoked GA, we estimated whether DAP impacted the AMPK/mTOR pathway in GA mice and THP‐1 macrophages. As depicted by western blotting, the p‐AMPK level was increased and the p‐mTOR level was decreased in mouse ankle tissues after MSU injection. Notably, DAP pretreatment prominently promoted AMPK phosphorylation and suppressed mTOR phosphorylation in the MSU + DAP groups compared with the MSU group (Figures 6A–C). Similar results were observed in MSU‐stimulated THP‐1 cells (Figures 6D–F), indicating that DAP promoted AMPK/mTOR signaling transduction in GA. To elucidate the mechanism by which DAP regulated AMPK/mTOR signaling, we evaluated the interaction between DAP and AMPK by molecular docking. The docking results show that DAP forms hydrogen bonds with the amino acid residues Glu‐96 and Val‐96 of AMPK (binding energy: −6.4 kcal/mol), indicating stable binding between DAP and AMPK (Figure S1).

### 3.7 Inhibition of AMPK signaling reverses DAP‐mediated effects on autophagy and inflammation in THP‐1 cells

**FIGURE 7:**
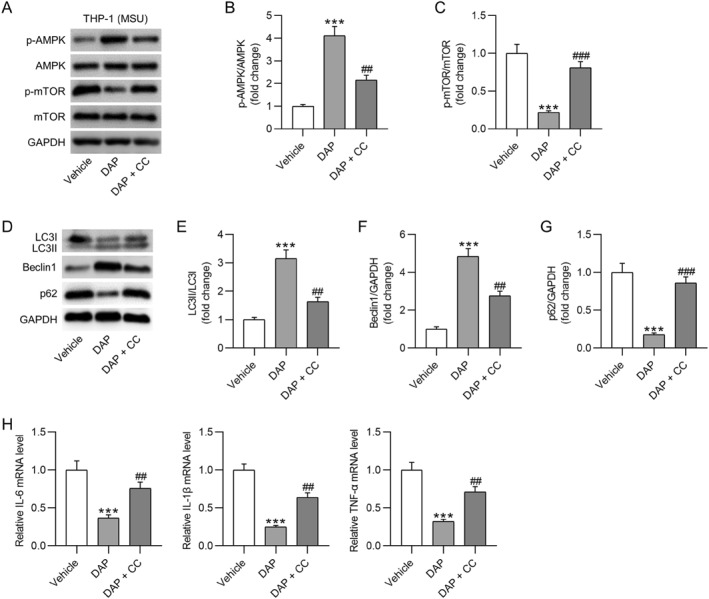
Inhibition of AMPK signaling reverses DAP‐mediated effects on autophagy and inflammation in THP‐1 cells. (A–C) Western blotting showing (p)‐AMPK and (p)‐mTOR protein expression in monosodium urate‐stimulated THP‐1 cells pretreated with DAP or DAP + CC (an AMPK inhibitor). (D–G) Western blotting for estimating autophagy‐related protein levels in indicated THP‐1 macrophages. (H) RT‐qPCR analysis of cytokine mRNA expression in THP‐1 cells. DAP, daphnetin. ***p < 0.001 versus vehicle; ##*p* < 0.01, ###*p* < 0.001 versus DAP.

Subsequently, we validated whether AMPK/mTOR signaling transduction was involved in DAP‐mediated effects on inflammation and autophagy in GA. THP‐1 cells were treated with CC, a selective AMPK inhibitor, to block signaling transduction. As expected, the p‐AMPK level was remarkably reduced, and the p‐mTOR level was elevated in MSU‐stimulated THP‐1 cells after CC treatment (Figures 7A–C). Moreover, we found that CC treatment markedly offset the increase in the LC3II/LC3I ratio and Beclin1 expression and the decrease in p62 expression induced by DAP in THP‐1 cells (MSU‐stimulated) (Figures 7D–G). In parallel, the DAP‐induced reduction in proinflammatory cytokine mRNA expression in MSU‐stimulated THP‐1 cells was prominently counteracted by the AMPK inhibitor (Figure 7H). Collectively, DAP relieved inflammation and activated autophagy in MSU‐stimulated THP‐1 cells by regulating AMPK/mTOR signaling transduction.

## 4 DISCUSSION

This study reveals that DAP pretreatment mitigated MSU injection‐induced joint swelling and synovial pathological changes in mice. Moreover, DAP preconditioning impeded inflammatory cytokine production and enhanced autophagy in both MSU‐induced GA mice and THP‐1 macrophages. Mechanistically, DAP activated AMPK signaling and inactivated mTOR signaling, and blocking AMPK signaling counteracted DAP‐mediated effects on inflammation and autophagy in THP‐1 cells.

MSU crystal deposition is the most basic clinical manifestation in GA.^24^ Based on previous evidence, we established a GA mouse model by injecting an MSU crystal suspension into the ankle joint. As expected, MSU‐injected mice showed significant joint swelling and synovial damage. Natural compounds have been recognized as potential therapeutic agents due to their beneficial effects, low toxicity, and high availability.^25^ DAP has been reported to have antiarthritic effects in osteoarthritis and collagen‐induced rheumatoid arthritis.^18^, ^20^ Similarly, our results revealed that DAP treatment prevented MSU‐induced joint swelling and pathological damage in the synovial tissues of the ankle joint, indicating its protective role in GA.

Macrophages are key effector cells in acute GA attacks.^26^ MSU crystal accumulation in joints and surrounding tissues results in the activation of tissue‐resident macrophages, which engulf MSU crystals and secrete inflammatory factors such as IL‐6 and TNF‐α.^27^ Elevated serum levels of IL‐6 and IL‐1β have been observed in patients with GA.^28^ Consistently, this study revealed that MSU markedly promoted the inflammatory response in the mouse model and THP‐1 cells, as evidenced by increased levels of proinflammatory factors (IL‐6, IL‐1β, and TNF‐α). Moreover, many reports have demonstrated that DAP plays an anti‐inflammatory role in various inflammatory diseases.^16^, ^17^ Similarly, our results displayed that DAP pretreatment could alleviate MSU‐triggered inflammation in both GA mice and THP‐1 macrophages.

Autophagy works as a critical regulator of inflammation. Under normal conditions, cells undergo autophagy to degrade and recycle dysfunctional proteins or organelles.^29^ Nonetheless, inadequate autophagy may lead to an excessive inflammatory response and cell death.^30^ Evidence suggests that autophagy is protective in several inflammatory diseases, including GA.^31^ LC3, Beclin1, and p62 are the most commonly studied autophagy‐related markers. During autophagy, LC3I is converted to LC3II, which is recruited to autophagosomal membranes.^32^ p62, also known as SQSTM1, is suppressed when autophagy is induced.^33^ Several studies have shown that LC3II/LC3I and Beclin1 levels are increased after MSU stimulation.^34^, ^35^ Consistent results were observed in this study. Moreover, previous evidence has indicated that DAP protects against liver failure by inhibiting the inflammatory response and inducing autophagy.^36^ This study depicted that DAP preconditioning prominently elevated the LC3II/LC3I ratio and Beclin1 expression and suppressed p62 expression in MSU‐induced GA mice and THP‐1 cells, confirming that DAP could enhance autophagy in GA.

AMPK is a key energy sensor that regulates cellular metabolism to maintain energy balance.^12^ AMPK activation leads to the suppression of mTOR, consequently activating autophagy in cellular processes.^37^ Of note, evidence suggests that AMPK/mTOR signaling‐mediated autophagy contributes to the amelioration of GA.^13^ Herein, elevated p‐AMPK levels were observed in the MSU‐induced model group compared to the control group, which is considered a compensatory response to MSU stimulation, thereby contributing to the alleviation of MSU‐induced inflammation and tissue damage. We found that DAP promoted AMPK activation and mTOR inactivation in MSU‐stimulated GA mice and THP‐1 macrophages. Furthermore, blocking AMPK signaling with a pharmacological inhibitor prominently counteracted DAP‐mediated suppression of inflammation and activation of autophagy in THP‐1 cells under MSU stimulation. A previous study has indicated that DAP induces cytoprotective autophagy in ovarian cancer by mediating AMPK/Akt/mTOR signaling,^38^ which partially supports our results. Additionally, considering the complexity of molecular mechanisms, further research is required to assess whether DAP protects against GA through other signaling pathways.

In conclusion, this study reveals the protective effect of DAP in MSU‐evoked acute GA in a mouse model and an in vitro cell model. The mechanism may be related to DAP‐mediated inflammation suppression and autophagy activation via AMPK/mTOR signaling transduction. Our findings may provide new ideas for preventing or treating GA.

## AUTHOR CONTRIBUTIONS

Zhiyong Liu conceived and designed the experiments. Zhiyong Liu, Aichun Chu, Zhiqian Bai and Chao Yang carried out the experiments. Zhiyong Liu, Aichun Chu, Zhiqian Bai and Chao Yang analyzed the data. Zhiyong Liu, Aichun Chu, Zhiqian Bai and Chao Yang drafted the manuscript. All authors agreed to be accountable for all aspects of the work. All authors have read and approved the final manuscript.

## CONFLICT OF INTEREST STATEMENT

The authors declare no conflicts of interest.

## ETHICS STATEMENT

All animal studies were conducted as per the Guide for the Care and Use of Laboratory Animals, with approval from the ethics committee of Wuhan Myhalic Biotechnology Co., Ltd (approval number: HLK‐202305397; approval date: May 26, 2024).

## Supporting information

Supporting Information S1

Figure S1

## Data Availability

The datasets used or analyzed during this study are available from the corresponding author upon reasonable request.
