# Supplementary material for: Daphnetin alleviates inflammation and promotes autophagy via the AMPK/mTOR pathway in gouty arthritis
Source: J Cell Commun Signal. 2025 Apr 28;19(2):e70011. doi: 10.1002/ccs3.70011 (PMC12037417; doi:10.1002/ccs3.70011)
Supplement: Supplementary file 1 — Supporting Information S1 [file CCS3-19-e70011-s001.docx]

Figure S1. Molecular docking of daphnetin (DAP) with AMPK.
